# A “safety cap” for improving hospital sanitation and reducing potential disease transmission

**DOI:** 10.1186/s12879-023-08566-5

**Published:** 2023-09-07

**Authors:** Lilong Liu, Yan Deng, Shouli Xia, Zengpeng Sun, Zhipeng Zhu, Weiyi Chen, Dongdong Xiao, Weiyong Sheng, Ke Chen

**Affiliations:** 1 Department of Urology, TongJi Hospital of TongJi Medical College, Huazhong University of Science and Technology, No. 1095 Jiefang Avenue, Wuhan, Hubei 430030 China; 2 Research Center for Tissue Engineering and Regenerative Medicine, Union Hospital, Tongji Medical College, Huazhong University of Science and Technology, Wuhan, China; 3 Department of Radiology, the First Affiliated Hospital of Wannan Medical College, Wuhu, China; 4 Department of Anesthesiology, Affiliated Drum Tower Hospital of Medical Department of Nanjing University, Nanjing, China; 5 School of Mechanical Engineering and Automation, Northeastern University, Shenyang, China; 6 Department of Emergency Surgery, Union Hospital, Tongji Medical College, Huazhong University of Science and Technology, Wuhan, China; 7 Department of Neurosurgery, Union Hospital, Tongji Medical College, Huazhong University of Science and Technology, No. 1277 Jiefang Avenue, Wuhan, Hubei 430022 China; 8 Department of Cardiothoracic Surgery, the First Affiliated Hospital of Wannan Medical College, No. 2 Zheshan West Road, Wuhu, Anhui 241001 China

**Keywords:** Respiratory secretions, Hospital sanitation, Pathogenic microorganisms, Potential diseases, Medical workers

## Abstract

**Background:**

During endotracheal intubation, extubation, tracheotomy, and tracheotomy tube replacement, the splashed airway secretions of patients will increase the risk of transmission of SARS-CoV‐2 and many other potential viral and bacterial diseases, such as influenza virus, adenovirus, respiratory syncytial virus, rhinovirus, Middle East respiratory coronavirus syndrome (MERS-CoV), *Streptococcus pneumoniae*, and *Mycobacterium tuberculosis*. Therefore, it is necessary to establish a barrier between patients and medical workers to reduce the risk of operators’ infection with potentially pathogenic microorganisms.

**Methods:**

We designed a “safety cap” that can be connected to the opening of an endotracheal tube or tracheotomy tube to reduce the diffusion area of respiratory secretions during the process of endotracheal intubation, extubation and tracheotomy tube replace, so as to reduce the infection risk of medical workers.

**Results:**

Through a series of hydrodynamic simulation analysis and experiments, we demonstrated that the use of “safety cap” can substantially limit the spatter of airway secretions, so as to improve the hospital sanitation.

**Conclusion:**

The “safety cap” can effectively limit the dissemination of patients’ respiratory secretions, thus reducing the risk of potential diseases transmission and may have certain application prospects.

## Introduction

The outbreak of severe acute respiratory syndrome coronavirus 2 (SARS-CoV-2) pandemic has made people pay more attention to the exposure risk of clinicians [1]. SARS-CoV-2 spreads through respiratory transmission by large droplets or fine aerosols expelled from patients and direct contact with patients or contaminated fomites [2], causing an infection rate of 3 ~ 11% among medical workers [3]. Large droplets or fine aerosols are usually generated from the respiratory tract of the infected patients during coughing, sneezing, speaking or during procedures such as sputum aspiration, endotracheal intubation, extubation, bronchoscopy, and tracheostomy [1, 4–6]. Recent studies have demonstrated that droplet generation tends to be accompanied by a multiphase turbulent gas (a puff) cloud that entrains ambient air and traps and carries clusters of droplets with a continuum of droplet sizes within it [7]. Large droplets (larger than 5 μm) can contaminate the nearby surface and spread viruses through direct and indirect contact (within 1 ~ 2 m), which is the main route of SARS-CoV-2 transmission [2, 8, 9].

**Fig. 1:**
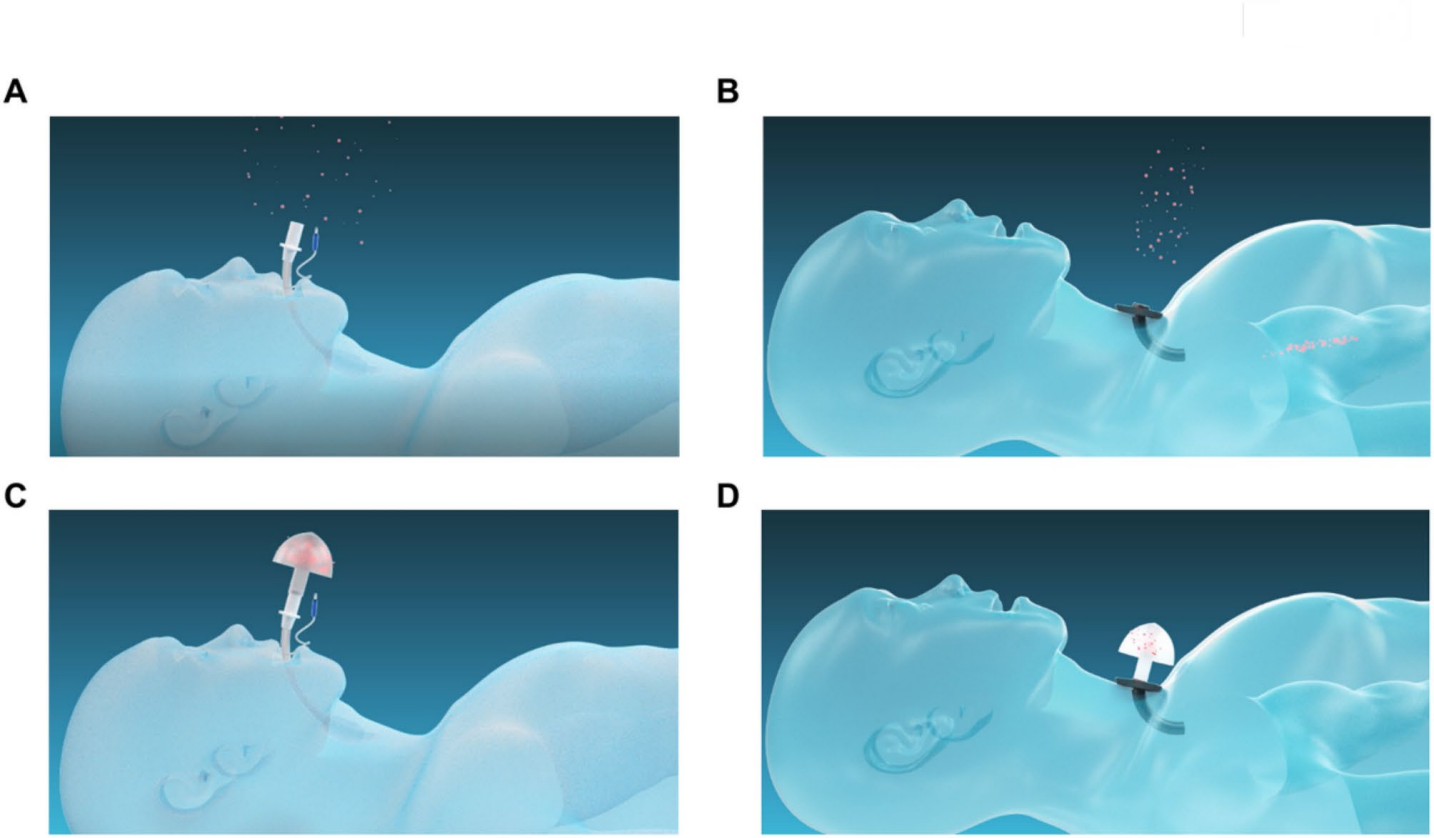
The purpose of inventing the “safety cap”. In the process of **(A)** endotracheal intubation, anesthesia resuscitation, and extubation, **(B)** tracheotomy and tracheotomy tube replacement, respiratory tract irritation may cause the patient to cough and splash large amounts of respiratory secretions (red particles). Design a “safety cap” that can be connected to the opening of the endotracheal tube **(C)** or tracheotomy tube **(D)** may help block the splashing of respiratory secretions when a patient cough

In addition to SARS-CoV-2, droplets may also carry microorganisms that spread other diseases [10], including influenza virus, parainfluenza viruses, adenovirus, respiratory syncytial virus, rhinovirus, measles virus, Middle East respiratory coronavirus syndrome (MERS-CoV), SARS-CoV, *Streptococcus pneumoniae*, Haemophilus influenzae, *Staphylococcus aureus*, *Mycobacterium tuberculosis*, *Bordetella pertussis*, *K. pneumoniae*, and *Pseudomonas spp* [8, 11–13]. In the process of endotracheal intubation, anesthesia resuscitation, extubation, tracheotomy, and regular replacement of tracheotomy tube (inner cannulas) in patients who have undergone a tracheostomy procedure, respiratory tract irritation may cause coughing, while the operator is directly exposed to the patient’s nose and mouth as well as the catheter opening [1, 14–19], which may cause a large amount of airway secretions to be sprayed on the operator’s face, hands and nearby surfaces [1], seriously increasing the risk of the operator to infect potentially pathogenic microorganisms (Fig. 1A, B). Therefore, it is urgent to establish a barrier between patients and medical workers to limit the diffusion area of the patient’s respiratory secretions, so as to avoid the contamination of nearby medical workers and surfaces by droplets from patients and reduce the risk of infection of the medical workers.

In this study, we designed a device called “safety cap” that can be connected to the opening of an endotracheal tube or tracheotomy tube. The objective is to prevent patients from spraying respiratory secretions directly to the operator during endotracheal intubation, extubation and tracheotomy tube replacement, so as to reduce the risk of infection of medical workers. Through a series of hydrodynamic simulation analysis and simulated patient cough experiments, we demonstrated the function of the “safety cap”.

## Materials and methods

### Structural composition and application scope of “safety cap”

**Fig. 2:**
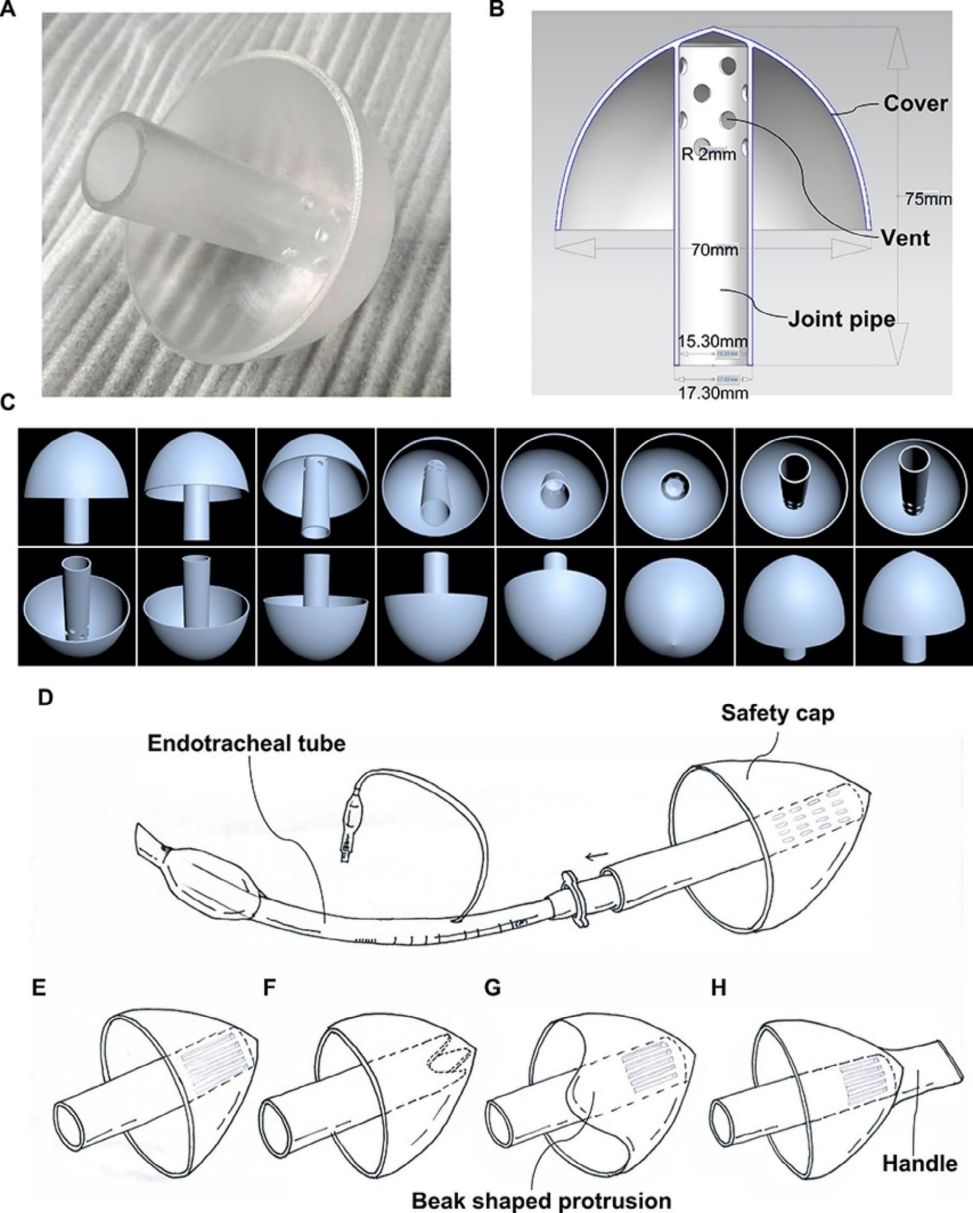
Structural composition of a “safety cap”. The “safety cap” consists of a joint pipe, several vents and a cover **(A-C)**. The joint pipe was used to connect the “safety cap” with the endotracheal tube or tracheotomy tube **(D)**. The second and the third designs of the “safety cap” with different types of vents **(E, F)**. Another type of “safety cap” with a beak-shaped protrusion **(G)**, the beak-shaped protrusion can be turned toward medical workers to increase the blocking effect on sprayed respiratory secretions. All the “safety cap” could be designed with a handle to facilitate removal from the endotracheal tube or tracheotomy tube **(H)**

To prevent medical workers from being contaminated by the respiratory secretions of the patients during endotracheal intubation, tracheal extubation, and tracheotomy tube replacement, we designed a “safety cap” that can be connected to the opening of the endotracheal tube or tracheotomy tube (Fig. 1C, D). As shown in Fig. 2A-C, the “safety cap” was composed of a joint pipe, several vents and a cover. The joint pipe was used to connect the “safety cap” with the endotracheal tube or tracheotomy tube. The vents were used for inspiration and expiration. The cover prevented respiratory secretions sprayed by patients through the endotracheal tube and the vents of the “safety cap”, which might contain viruses or other disease-causing microorganisms (Fig. 2D). The “safety cap” has 16 vents with a total area of 200.96 mm^2^, while the opening area of the endotracheal tube was 183.76 mm^2^. There were the second and third designs of the “safety cap” with different types of vents (Fig. 2E F). Another type of “safety cap” with a beak-shaped protrusion was shown in Fig. 2G, the beak-shaped protrusion can be turned toward medical workers to increase the blocking effect on sprayed respiratory secretions by using this type of “safety cap”. In addition, these “safety cap” could be designed with a handle to facilitate removal from the endotracheal tube or tracheotomy tube (Fig. 2H).

### Hydrodynamic simulation analysis of the “safety cap”

To determine whether the use of “safety cap” would increase the respiratory resistance of patients, we performed a hydrodynamic simulation analysis. In the DM design module of ANSYS Workbench Release 14 (ANSYS Corporation), the flow calculation area of endotracheal tube and the “safety cap” was obtained through Boolean operation. The flow rate at the inlet of endotracheal tube and the “safety cap” was set to 60 L/min according to a previous report [20], and the outlet pressure was set to 0 Pa. The reference pressure of each calculation area was set to 1 atmosphere (ATM), and the inner wall was set as a no-slip wall. The air density was 1.139 kg/m^3^, and the viscosity was 1.894e-05 Pa·s at 37 ℃. Computational fluid dynamics (CFD) calculations were performed using CFX software Release 14 (ANSYS Corporation) with k-Epsilon turbulence mode [21], and the convergence accuracy was set to 1e-4.

**Fig. 3:**
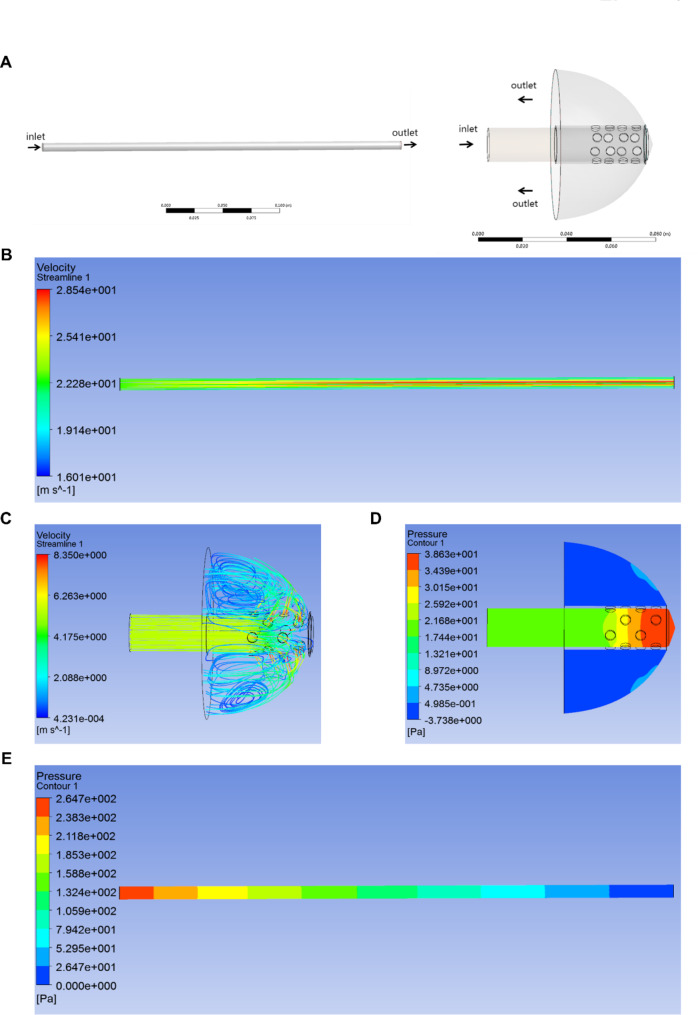
Hydrodynamic simulation analysis of the “safety cap”. The flow calculation area of endotracheal tube and “safety cap” **(A)**. The streamlined distribution of the computational domain in the endotracheal tube and “safety cap” **(B, C)**. The pressure cloud diagram in the calculation domain of “safety cap” and endotracheal tube **(D, E)**, from the inlet to the outlet of the “safety cap”, the pressure drop was approximately 21.4 Pa

The flow calculation area of endotracheal tube and “safety cap” was shown in Fig. 3A. Figure 3B C showed the streamlined distribution of the computational domain in the endotracheal tube and “safety cap”. The exhaled air flows out from the vents and impacts the inner surface of the cover, forming a low-speed vortex flow, thus preventing the respiratory secretions from directly splashing onto medical workers. After the patient’s exhaled air entered the endotracheal tube, the pressure in the endotracheal tube gradually decreased approximately 265 Pa from the inlet to the outlet. The pressure rose sharply when the gas reached the top area of the joint pipe, and then the pressure droped rapidly to approximately atmospheric pressure after the gas exited the vents. From the inlet to the outlet of the “safety cap”, the pressure drop was approximately 21.4 Pa (Fig. 3D, E). According to the calculation, since the total area of the vents was significantly larger than the cross-sectional area of the endotracheal tube (200.96 mm^2^ vs. 183.76 mm^2^), the “safety cap” only increased the flow resistance by about 8%; that is, the use of “safety cap” may not significantly affect the patient’s breathing.

### Simulation experiments of cough-splash respiratory secretions

A simulation experiment was conducted to investigate the splash-blocking effect of the “safety cap” on respiratory secretions sprayed by patients. A disposable pulse irrigator (APEXPULSETM, Apex (Guangzhou) Tools & Orthopedics Co., Ltd.) connected to the connecting tube was used to simulate the patient’s cough. It should be noted that the function of this disposable pulse irrigator is to spray liquid/air at a rate of 900 ± 300 mL/min in a pulse, without inhalation. The secretion in the patient’s respiratory tract was simulated using a phosphor suspension or *Escherichia coli* (*E. coli*) with an ampicillin resistance gene (Vigene Biology, China) in the connecting tube. An acrylic cover on the patient’s head, neck and chest was used to simulate the medical workers around the patient, and then the protective effect of the “safety cap” was explored through the following experiments. Firstly, we placed an endotracheal tube with or without a “safety cap” at the opening through the mouth of a mannequin, and the front end of the endotracheal tube was connected with a disposable pulse irrigator through the connecting tube. Next, the acrylic cover was placed on the patient’s head, neck and chest. Then, a liquid storage bag containing 20 mL of phosphor suspension or *E. coli* suspension (the concentration of bacteria was 10^6^ colony-forming units (CFU)/mL) was connected to the connecting tube; the control group received 20 mL of sterile PBS. Subsequently, the phosphor suspension, *E. coli* suspension or sterile PBS was sprayed through the liquid storage bag-connecting tube and endotracheal tube in the form of a pulse for 30 s through the disposable pulse irrigator. Then, fluorescent pictures were taken on the acrylic cover under dark conditions, and samples were collected at the positions marked in advance on the acrylic cover and the chest of the mannequin for subsequent quantitative analysis of *E. coli*.

### Escherichia coli culture

All the samples taken from the acrylic cover and the chest of the mannequin were inoculated into 20 mL of Luria Bertani (LB) broth medium (NaCl 10 g/L, peptone 10 g/L, and yeast extract 5 g/L) with a final ampicillin concentration of 100 µg/mL and then incubated at 37 °C and 150 rpm for 18 h. After the incubation period, the growth of bacteria was evaluated by measuring the absorbance at 600 nm and the turbidity of the culture medium. Quantification of live *E. coli* was performed by serially diluting the bacterial culture medium in PBS solution and then inoculating it on LB agar plates. CFU were calculated after incubation for 18–24 h at 37 °C.

### Statistical analysis

GraphPad Prism 6.02 (GraphPad Software, Inc., San Diego) was used for statistical analysis. Unpaired Student’s T tests were used for comparisons between groups. Data were expressed as the mean ± standard deviation (SD). A value of P < 0.05 was considered statistically significant. *P < 0.05, **P < 0.01, ***P < 0.001, ****P < 0.0001.

## Results and discussions

### Cough-splash simulation experiment with a phosphor suspension simulating respiratory secretions

**Fig. 4:**
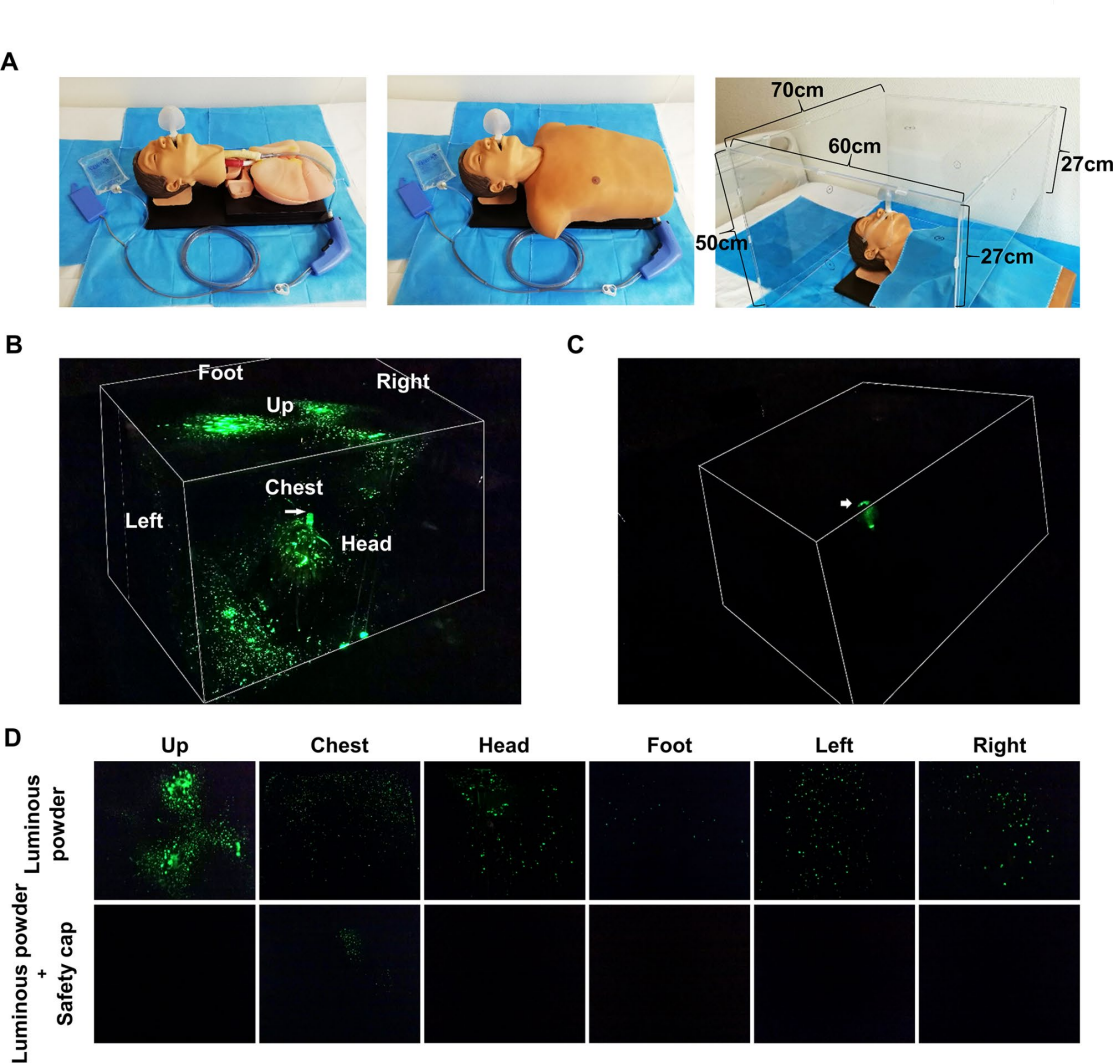
Cough-splash simulation experiments with a phosphor suspension simulating respiratory secretions. **(A)** Operation diagram of simulation experiments, the disposable pulse irrigator connected to the connecting tube was used to simulate the patient’s cough, the secretion in the patient’s respiratory tract was simulated using a phosphor suspension in the connecting tube, an acrylic cover on the patient’s head, neck and chest was used to simulate the medical workers around the patient. It should be noted that during the experiment, the tip of the endotracheal tube needs to pass through the trachea carina of the simulated human to connect with the pipeline of the disposable pulse irrigator, so the depth of the endotracheal tube is beyond the normal range in the experiment. **(B-D)** The distribution of the patient’s respiratory secretions (phosphor suspension) around the patient with or without the “safety cap”. The experiment was repeated three times

As shown in Fig. 4A, we performed a simulation experiment to investigate the blocking effect of the “safety cap” on respiratory secretions sprayed by patients. In the absence of a “safety cap”, the secretion (phosphor suspension) of the patient’s respiratory tract was sprayed in all directions through the endotracheal tube when coughing, with the highest concentration was directly above the endotracheal tube opening, followed by the cephalic, left and right sides of the patient, and the concentration was lower in the chest and feet sides. After using a “safety cap”, the spraying range of respiratory secretions during coughing was significantly reduced, only a small amount of fluorescence was detected on the chest of the patient, with no fluorescence was detected in other sides (Fig. 4B-D). These results indicate that the use of the “safety cap” can significantly limit the spraying range of respiratory secretions and reduce the risk of potential diseases transmission.

### Cough-splash simulation experiment with *E. coli* suspension simulating respiratory secretions

**Fig. 5:**
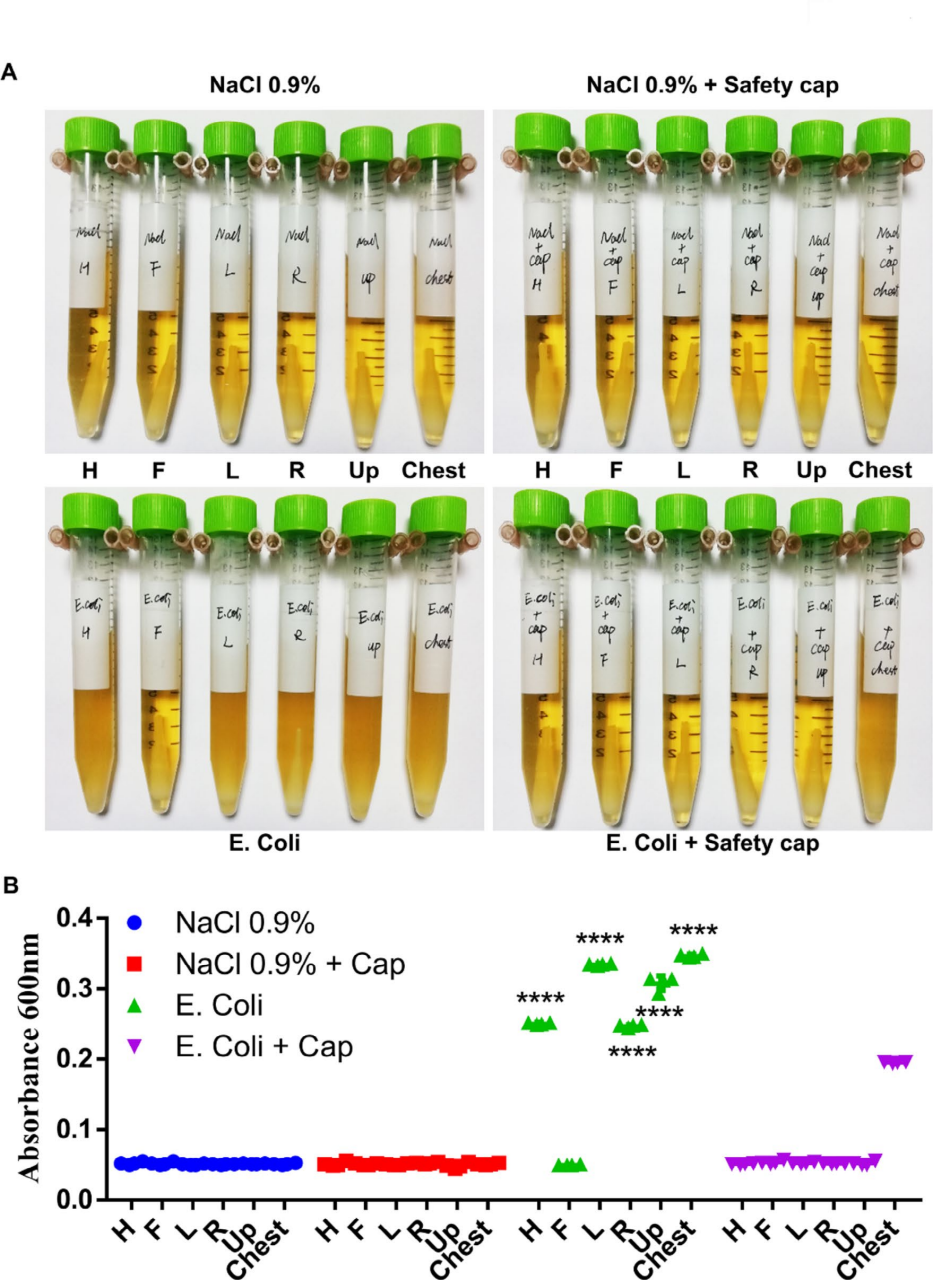
Cough-splash simulation experiments with *E. coli*suspension simulating respiratory secretions. All samples collected from pre labelled positions on the acrylic cover and the chest of the mannequin were inoculated into LB broth medium **(A)**. After incubation for 18 h, the absorbance at 600 nm was detected, n = 5 **(B)**. The experiment was repeated three times. Data were expressed as the mean ± SD. A value of P < 0.05 was considered statistically significant. *P < 0.05, **P < 0.01, ***P < 0.001, ****P < 0.0001

**Fig. 6:**
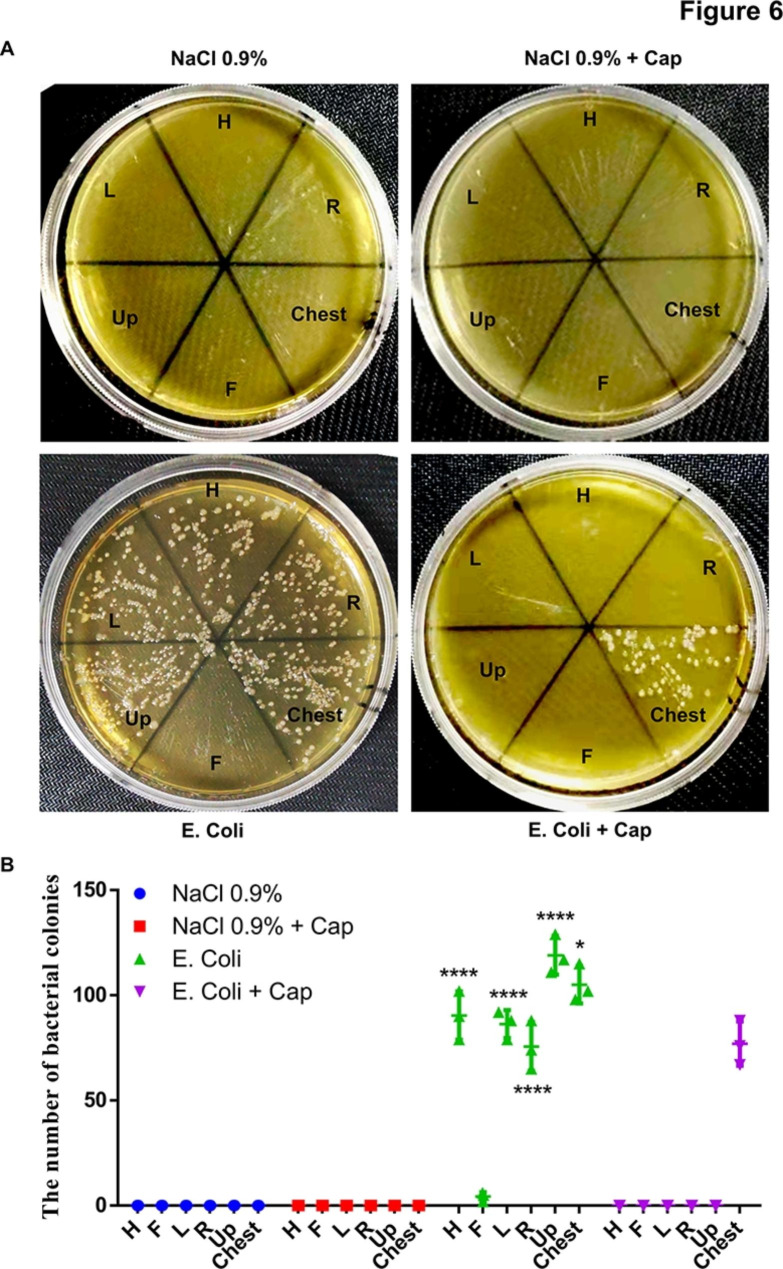
Quantification of live ***E. coli*** in samples collected from pre labelled positions on the acrylic cover and the chest of the mannequin. All samples were inoculated into LB agar plates **(A)**. **(B)** Comparison of the number of *E. coli* colonies among different groups, n = 3. The experiment was repeated three times. Data were expressed as the mean ± SD. A value of P < 0.05 was considered statistically significant. *P < 0.05, **P < 0.01, ***P < 0.001, ****P < 0.0001

Subsequently, *E. coli* suspension was used to simulate the secretions in the respiratory tract of patients, and the cough-splash simulation experiment was carried out. All samples collected from pre labelled positions on the acrylic cover and the chest of the mannequin were inoculated into LB broth medium and LB agar plates. As shown in Fig. 5A and B, in the absence of “safety cap”, in addition to the patient’s foot side, a large number of *E. coli* were present in samples collected on the cephalic side, above the tracheal tube opening, the chest, and the left and right sides of the patient. However, when using a “safety cap”, *E. coli* was found only in samples collected from the patient’s chest. Notably, samples collected from the patient’s chest when using the “safety cap” contained significantly less bacteria than those without the “safety cap”. In addition, as shown in Fig. 6A, B, results showed that if there was no “safety cap”, respiratory secretions would be sprayed in all directions except the patient’s feet side when coughing. However, the use of “safety cap” limited the respiratory secretions sprayed by the patients to the patients’ chest and significantly reduced the composition of the sprayed secretions, indicating that the use of “safety cap” could significantly reduce the risk of potential diseases transmission.

### Several other “safety cap” designs

**Fig. 7:**
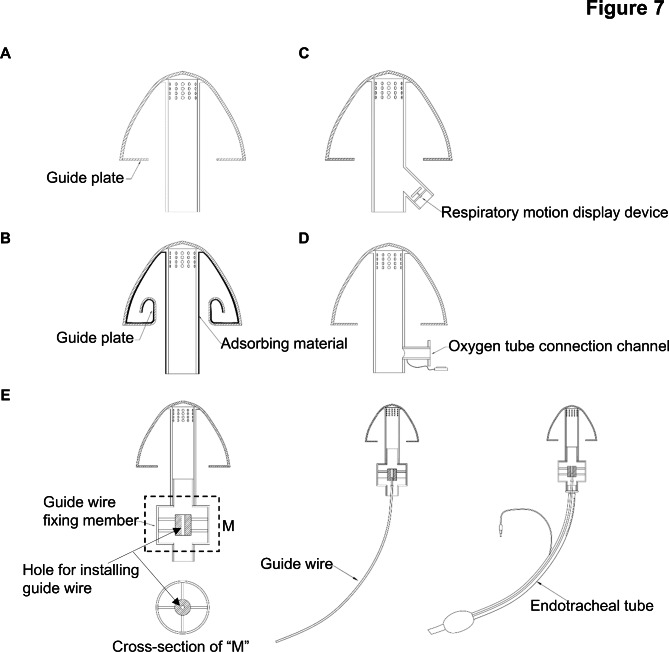
Several other “safety cap” designs. A gas guide plate was added to further limit the airflow velocity of cough splash **(A)**. The adding of adsorption material on the inner surface of the cover might adsorb the respiratory secretions of patients and further reduce the risk of potential diseases transmission **(B)**. A side branch pipe was additionally provided on the joint pipe, which could be used to connect the respiratory motion display device **(C)** or the oxygen tube **(D)**. A guide wire fixing member can be installed in the joint pipe to connect the guide wire to facilitate the formation and placement of an endotracheal tube during endotracheal intubation **(E)**

To further improve the function and protection effect of the “safety cap”, we reformed its structure. A gas guide plate was added to further limit the airflow velocity of cough splash (Fig. 7A, B). The adding of adsorption material on the inner surface of the cover might adsorb the respiratory secretions of patients and further reduce the risk of potential diseases transmission (Fig. 7B). A side branch pipe was additionally provided on the joint pipe, which could be used to connect the respiratory motion display device (Fig. 7C) or the oxygen tube (Fig. 7D). In addition, a guide wire fixing member with a hole for installing guide wire can be installed in the joint pipe to connect the guide wire to facilitate the formation and placement of an endotracheal tube during endotracheal intubation (Fig. 7E).

## Discussions

It was found that SARS-CoV‐2 viral in sputum and upper respiratory secretions was the highest [22], which caused the world’s attention to reduce the occupational safety of medical workers. In the process of endotracheal intubation, anesthesia resuscitation, extubation, tracheotomy, and tracheotomy tube replacement, medical workers may be infected with various viruses and bacteria other than SARS-CoV-2 [10], which seriously threatens the health of medical workers.

To reduce viral exposure during sputum aspiration, endotracheal intubation, bronchoscopy, and tracheostomy, efforts have been made to standardize clinical procedures and develop personal protective equipments [5]. For example, some scholars suggested that medical workers should use 5 min of preoxygenation with 100% oxygen, rapid sequence induction techniques, and small tidal volume manual ventilation to avoid potential aerosolization of the virus from patient’s airways [23, 24]. An “aerosol box”, consisting of a transparent plastic cube designed to cover the patient’s head, contains two circular ports through which the doctor’s hands perform the airway procedures, reportedly effectively blocking the spatter of the patient’s airway secretions [6, 25, 26]. In addition, a sufficiently large (> 100 cm × 100 cm is recommended) PVC membrane with a hole (sealed when necessary) in the center for the connection between the face mask and the circuit has been reported to similarly control the source of infection and enhance the protective measures of medical workers [1]. During the epidemic, all of the above measures played a role in protecting medical workers in around the world. However, after the epidemic is under control, the complicated and costly protective measures are not applicable. Therefore, it is necessary to develop an airway secretion blocking device that is simple to operate, easy to remove after use without contaminating the users and suitable for reducing the transmission risk of potential diseases.

Direct contact with large droplets from infected persons and contaminated fomites is considered to be the main route of transmission of respiratory viruses [8]. A large number of studies on the distance of horizontal droplet showed that the propagation distance of droplets was more than 2 m, or even more than 8 m in some cases [9, 27]. In this study, we designed a series of “safety cap” that can be connected to the opening of endotracheal tube or tracheotomy tube. The cough-splash simulation experiments using phosphor suspension or *E. coli* suspension to simulate respiratory secretions showed that the use of “safety cap” can significantly limit the spraying range of respiratory secretions and significantly reduce the component of sprayed secretions, indicating that “safety cap” can significantly improve the hospital sanitation and reduce the transmission risk of various pathogens carried by respiratory secretions.

Clinical practice has proven the effectiveness of bacteria/virus filters in limiting the transmission of respiratory microorganisms, but we believe that the bacterial filter will greatly increase the respiratory resistance of patients and may not be suitable for patients who have undergone a tracheostomy procedure and need to replace tracheotomy tube (inner cannulas), as well as for patients who are being extubated and waiting for spontaneous breathing recovery. Through this study, we provide an alternative protective solution. In this study, our main objective was to investigate the effectiveness of using a “safety cap” to block respiratory secretions expelled during endotracheal intubation, extubation (including the process of waiting for patients to regain sufficient strength for autonomous breathing), and regular replacement of tracheotomy tube (inner cannulas) in patients who have undergone a tracheostomy procedure, thereby preventing direct contamination (Respiratory secretions are directly sprayed onto medical workers) of medical workers’ faces and bodies by patient respiratory secretions. We believe that no medical workers would want patient respiratory secretions sprayed onto their face. Although the use of a “safety cap” may not completely prevent disease transmission caused by aerosol leakage, it can significantly reduce direct exposure of medical workers’ faces, clothing, and skin to patient respiratory secretions, thus improving the hospital sanitation and reducing (rather than eliminating) potential risks associated with disease transmission.

The “safety cap” has advantages of low cost, simple operation, and a good protective effect. Based on this study, we propose that “safety cap” may be applicable to the following 3 clinical procedures that are prone to causing patient coughing. However, the practicality and effectiveness of limiting airway secretion spread in these use cases still require thorough research for further validation [1]. Before endotracheal intubation, the “safety cap” can be connected to the endotracheal tube opening to prevent the respiratory secretions emitted by patients when coughing and to prevent pollution to the medical workers [2]. Before extubation, the “safety cap” can be connected to the endotracheal tube opening to block the airway secretions, which may carry pathogenic microorganisms when the patient coughs [3]. In the process of tracheotomy tube replacement, connecting the “safety cap” to the tracheotomy tube opening may effectively block the respiratory secretions sprayed by patients, and effectively reduce the occupational exposure risk of medical workers. It is essential to emphasize that these potential clinical applications are based on the results of simulated experiments and will require further validation through future clinical trials.

However, this study still has certain limitations, as the “disposable pulse irrigator” sprays liquid/air at a rate of 900 ± 300 mL/min in a pulse, it is not a true cough, since the speed of cough flow can be as high as 300 L/min. Additionally, the patients undergoing intubation usually receive sedation and paralytics, which suppress coughing and spontaneous breathing. Once the endotracheal tube is inserted and connected to a ventilator, the exposure time to the patient’s lower airway is very short, resulting in a low risk of generating aerosols, especially bioaerosols [28]. Placing the “safety cap” on the endotracheal tube during insertion may hinder visibility and increase weight, potentially making it more difficult to manipulate and pass the tube through the vocal cords. The secretions in the oral cavity and trachea can still be expectorated through the gap between the endotracheal tube and the trachea if the cuff is not inflated. Furthermore, extubation may generate some aerosols; however, when patients are ready for extubation, the risk of transmission from exhaled aerosols is very low due to resolved or improved infection. Therefore, the exact role of “safety cap” is to prevent patients from spraying respiratory secretions directly to the operator during airway management, especially in the process of replacing the tracheotomy tube in patients who had undergone tracheotomy, improve the hospital sanitation and reduce the transmission risk of potential diseases.

## Conclusion

During the process of endotracheal intubation, anesthesia resuscitation, extubation, tracheotomy, and tracheotomy tube replacement, the airway secretions spattered by patients coughing will increase the transmission risk of many potential viral and bacterial diseases. Thus, it is necessary and meaningful to establish a barrier between patients and medical workers to reduce the risk of operator infection with potentially pathogenic microorganisms. Here, we adopted a simulation experiment to confirm that our “safety cap” have certain application prospects, as they could effectively prevent patients from spraying respiratory secretions directly to the operator during endotracheal intubation, extubation and tracheotomy tube replacement, thus reducing the risk of potential diseases transmission. However, the above expectations are based on the results of simulation experiments, and more experimental verification and publicity are needed to promote the clinical application of these “safety cap”.

## Data Availability

All data generated or analysed during this study are included in this published article.
